# The impact of long-term care insurance in China on beneficiaries and caregivers: A systematic review

**DOI:** 10.52872/001c.29559

**Published:** 2021-11-16

**Authors:** Simiao Chen, Linye Li, Juntao Yang, Lirui Jiao, Todd Golden, Zhuoran Wang, Haitao Liu, Peixin Wu, Till Bärnighausen, Pascal Geldsetzer, Chen Wang

**Affiliations:** 1 Heidelberg Institute of Global Health (HIGH), Faculty of Medicine and University Hospital, Heidelberg University; Chinese Academy of Medical Sciences and Peking Union Medical College; 2 Chinese Academy of Social Sciences; 3 State Key Laboratory of Medical Molecular Biology, Institute of Basic Medical Sciences, Chinese Academy of Medical Sciences & Peking Union Medical College; 4 Columbia University; 5 Division of Cancer Control and Population Sciences, National Cancer Institute; 6 Chinese Academy of Medical Sciences and Peking Union Medical College; 7 Heidelberg Institute of Global Health (HIGH), Faculty of Medicine and University Hospital, Heidelberg University; Chinese Academy of Medical Sciences and Peking Union Medical College; Department of Global Health and Population, Harvard School of Public Health; 8 Heidelberg Institute of Global Health (HIGH), Faculty of Medicine and University Hospital, Heidelberg University; Division of Primary Care and Population Health, Department of Medicine, Stanford University; 9 Chinese Academy of Medical Sciences and Peking Union Medical College; National Clinical Research Center for Respiratory Diseases, Beijing, China; Department of Pulmonary and Critical Care Medicine, Center of Respiratory Medicine, China-Japan Friendship Hospital

**Keywords:** long-term care insurance, systematic review, China, beneficiaries and caregivers

## Abstract

**Background:**

China’s long-term care insurance (LTCI) policy has been minimally evaluated. This systematic review aimed to assess the impact of China’s LTCI pilot on beneficiaries and their caregivers.

**Methods:**

This review is based on a search of peer-reviewed studies in English (Embase, MEDLINE, Web of Science) and Chinese (China National Knowledge Infrastructure [CNKI], VIP, Wanfang) databases from January 2016 through July 2020, with all studies published in English or Chinese included. We included quantitative analyses of beneficiary-level data that assessed the impact of LTCI on beneficiaries and their caregivers, with no restriction placed on the outcomes studied.

**Results:**

Nine studies met our inclusion criteria. One study was a randomised trial and two used quasi-experimental approaches. Four studies examined LTCI’s effect on beneficiaries’ quality of life, physical pain, and health service utilisation; one study reported the effect on beneficiaries’ healthcare expenditures; and one study evaluated the impact on caregivers’ care tasks. These studies generally found LTCI to be associated with an improvement in patients’ quality of life (including decreased physical pain), a reduction in the number of outpatient visits and hospitalisations, decreased patient-level health expenditures (e.g. one study reported a reduction in the length of stay, inpatient expenditures, and health insurance expenditures in tertiary hospitals by 41.0%, 17.7%, and 11.4%, respectively), and reduced informal care tasks for caregivers. In addition, four out of four studies that evaluated this outcome found that beneficiaries’ overall satisfaction with LTCI was high.

**Conclusion:**

The current evidence base for the effects of LTCI in China on beneficiaries and their caregivers is sparse. Nonetheless, the existing studies suggest that LTCI has positive effects on beneficiaries and their caregivers. Further rigorous research on the impacts of LTCI in China is needed to inform the future expansion of the program.

## INTRODUCTION

China is facing a rapidly ageing population, which poses challenges for its healthcare system and society at large. In 2015, there were 143.86 million people aged 65 years or older in China, representing 10.5% of the population. Just four years later, by 2019, this age group had expanded to 176.03 million people, 12.6% of the population.^1,2^ By 2050, the population over age 65 is expected to reach 366 million, over a quarter of the population (26.1%), with the number of people aged 80 years or older being the fastest-growing group (increasing from ~22 million [1.5%] in 2015 to 115 million [8.2%] in 2050).^3^

Alongside the rapid growth of the older adult population in China, the number of people with debilitating comorbidities is also increasing, from 15.63 million in 2015 to 40 million by 2060.^4,5^ Non-communicable diseases (NCDs) represent the majority of these comorbidities, including stroke, cardiovascular disease, diabetes, hypertension, Alzheimer’s disease or related dementias and multiple comorbidities,^6–11^ which result in limitations in their daily activities. Therefore the need for long-term care in China is expected to increase rapidly and substantially.^4^

Family members and other informal (unpaid) caregivers in China currently assume most care for older adults and older disabled adults,^12–14^ including the physical, psychological, and financial aspects. Their adult children, spouses, relatives, and friends most often provide informal care; formal care is usually provided by long-term care workers and health professionals (e.g. nurses, therapists, and physicians who are available to provide skilled nursing, rehabilitation, and medical services).^15^ Informal care is most common,^16,17^ yet frequently results in substantial psychological and health consequences, and employment and income effects to the caregivers.^18,19^ Given this, health insurance, particularly long-term care insurance (LTCI), has been receiving increasing attention.^20–23^ The World Health Organization (WHO) considers LCTI a promising means of achieving universal healthcare coverage^24^ and suggested China initiate an LTCI policy to support disabled and older adults’ increasing demand for basic life care and daily nursing services.^25–28^

In 2016, China issued guidelines on implementing a LTCI policy and officially piloted LTCI in 15 cities.^29^ LTCI is often termed a “sixth social insurance” in addition to China’s “five social insurances system” (pension insurance, medical insurance, work-related injury insurance, unemployment insurance, and childbirth insurance). LTCI aims to provide disabled and older adults with affordable basic services (mainly basic life care services and basic nursing services) and allows participants to purchase specialised nursing services from the private sector.^30^ As of June 2019, China’s LTCI pilot program covered 88.54 million people, with 426,000 people receiving LCTI benefits.^31^ In 2020, China expanded the LTCI pilot to an additional 14 cities.^32^

The impact of LTCI in China on beneficiaries and caregivers is still unclear. Quantitative evidence on the impact of LTCI in China is crucial to improving the design and future expansion of LTCI in China and could also provide important lessons for other countries facing similar demographic changes. To address these gaps, we conducted the first systematic review of the impact of China’s LTCI on (i) health status; (ii) utilisation of healthcare services; (iii) health expenditures; (iv) healthcare quality; and (v) user satisfaction, following the PRISMA checklist (see Appendix 2).^33^

## METHODS

### ELIGIBILITY CRITERIA

All peer-reviewed studies published in English or Chinese were included, which reported data from 2016 onwards (to coincide with the inception of the LTCI pilot in China) and contained a quantitative analysis of individual-level data that used experimental, quasi-experimental, or multivariable regression methods. We excluded modeling studies, ecological studies, and studies with a sample size less than 30. However, we did not restrict the outcomes examined.

### INFORMATION SOURCES AND SEARCH STRATEGY

We searched English (Embase, MEDLINE, Web of Science) and Chinese (China National Knowledge Infrastructure [CNKI], VIP, Wanfang) databases from January 2016 through July 2020, using the search terms {“impact” or “association” or “effect”}, {“long-term care insurance” or “long term care insurance”, “insurance, long term care”, “care insurance”} and “China.” (See Appendix for the full search strategy, terms, and outcomes.)

### SELECTION AND DATA COLLECTION

Two reviewers (JY and ZW) independently screened titles and abstracts in English and Chinese, using the search terms. Two additional authors (SC and LL) read the full text of all identified articles and selected the final manuscripts for inclusion. Full-text copies of potentially relevant articles were examined, and their reference lists were reviewed for additional pertinent publications.

### BIAS ASSESSMENT AND CERTAINTY ASSESSMENT

The risk of bias for each dataset was assessed using the component approach adopted by the Cochrane Collaboration.^34^ Two reviewers (SC and LL) assessed each study independently. If there was disagreement, they consulted with two additional authors (PG and TB) to establish a consensus on the final inclusion.

### SYNTHESIS METHODS

The studies were synthesised into a table by design, setting, population, size, and outcome, Table 1. Additionally, a summary of each study’s findings and an assessment of their evidence quality was tabulated, Table 2.

## RESULTS

### STUDY SELECTION

The Chinese-language database search identified 5,179 titles and abstracts, with 2,635 unique records after removing duplicates, with 13 of these retained for full-text review. The English search identified 797 titles and abstracts, with 715 unique records after removing duplicates, with 11 of these retained for full-text review. After excluding modeling studies, ecological studies, and studies with sample size <30, 6 Chinese language studies and 3 English language studies were selected for this review.

### STUDY CHARACTERISTICS

Table 1 summarises the nine included studies on the impact of long-term care insurance. One study is intentionally listed twice as it investigated both health-related outcomes and health expenditures. Four studies reported on health (improving patients’ quality of life, reducing the number of outpatient visits and the average hospitalisation frequency, improving mental health and relieving physical pain) and one study reported on health expenditures (reducing the disabled older adults’ health expenses). Four studies reported on satisfaction (the beneficiaries’ overall satisfaction is high) and one study reported on informal care (reducing caregivers’ household activities of daily living tasks [HDL], activities of daily living tasks [ADL], instrumental activities of daily living tasks [IADL] and supervision tasks). Most studies (n=6) was based on data collected in 2018 and 2019—with all published in 2019 and 2020, and the most commonly represented provinces/cities were Shanghai (n=6) and Hubei (n=2). Five studies relied on data from interview-based and questionnaire-based surveys. Two studies adopted the difference-in-difference method and sampling strategies were generally well described.

### QUALITY OF INCLUDED STUDIES

All studies were assessed on the rigor of study design, completeness of data, definition of intervention group and control group, statistical analysis and adjustment for confounding.^34^ Each quality criterion was classified as low, medium, or high risk of bias for each dataset according to its method, sample size and study period. Regression analysis only measures association and might omit variables or be affected by other confounders, which might result in coefficient bias. Thus, this method is not aimed at causal relationship analysis and was categorized as low quality. The difference-in-differences method eliminates some confounders and is more rigorous for causal relationship analysis compared to regression analysis, thus this method was considered medium quality. Randomized controlled trials were considered the most effective method to examine causal relationships and were rated high evidence quality. Studies with a large sample size were rated as higher quality. All the studies occurred during the LTCI pilot program and were assessed as having no difference in terms of study period.

Based on these comprehensive criteria, six studies which all used regression analysis and had a sample size less than 10,000 participants were rated low evidence quality, including Qi et al. (2019),^37^ Zhang et al. (2020),^39^ Zhang et al. (2019),^40^ Chen et al. (2020),^41^ Dai et al. (2019),^42^ and Zhang (2019).^43^ Two studies, Feng et al. (2020)^36^ and Ma et al. (2019),^38^ were classified as having medium evidence quality as they utilized a difference-in-differences design and had a sample size greater than 10,000 participants. Only Yu et al. (2020),^35^ a randomized controlled trial, was classified as having high evidence quality.

### CHINA’S LONG-TERM CARE INSURANCE AND HEALTH

Overall, LTCI was associated with greater access to healthcare services and better health for the beneficiaries. According to a national-level survey of older adults (China Health and Retirement Longitudinal Study), conducted by Ma et al. (2019),^38^ and a city-level survey of older stroke patients in Shanghai, conducted by Yu et al. (2020),^35^ the introduction of LTCI was associated with positive effects (e.g. improving older stroke patients’ survival quality) on health conditions, and was correlated with a reduction in middle-aged and older adults’ number of outpatient visits as well as average hospitalisation frequency. These results were also supported by another study, Feng et al. (2020),^35^ which found that LTCI was associated with a reduction in the beneficiaries’ length of stay. Ma et al. (2019)^38^ also found that LTCI was correlated with improvement of the beneficiaries’ mental health status and relief of their physical pain without negative consequences.

### IMPACT OF LONG-TERM CARE INSURANCE ON HEALTHCARE UTILISATION AND EXPENDITURES

Three studies all found that the implementation of LTCI was associated with a reduction in healthcare utilisation and expenditures, which could be regarded as an effective means of alleviating older adults’ financial pressure and protecting the households against impoverishment from out-of-pocket expenditures. Qi et al. (2019)^37^ found that the enrollment of older adults in LTCI in general was related with out-of-pocket medical expenses while two other studies were more detailed. Feng et al. (2020)^36^ found that a 1-yuan increase in LTCI expenditure would generate an 8.6-yuan decline in health insurance expenditures and an 8.1% monthly decrease in outpatient visits in tertiary hospitals among people aged 80 years and above. Ma et al. (2019)^38^ also found that the implementation of LTCI was associated with a reduction in the average outpatient expenses of the middle-aged and older adults in the targeted pilot city, Qingdao, by 210.51 yuan per month and a reduction in the average hospitalisation expenses by 1,901.69 yuan per year.

### IMPACT OF LONG-TERM CARE INSURANCE ON INFORMAL CARE

Zhang et al. (2020)^39^ conducted an interview-based survey to study the impact of LTCI on informal care in Shanghai. LTCI was associated with a reduction of a weekly average of 12.36 hours of informal care (including household activities of daily living [HDL] tasks, activities of daily living [ADL] tasks, instrumental activities of daily living [IADL] tasks and supervision tasks) in 407 families. The study also found that although the reduction of total informal care time varied according to care recipients’ gender and health status, each additional hour of formal care generally reduced informal care by 0.473 hours. Another city-level survey, conducted by Yu et al. (2020),^35^ of older stroke patients in Shanghai, also found that the introduction of LTCI was correlated with a decrease in caregivers’ burden.

### LONG-TERM CARE INSURANCE SATISFACTION

Overall, these studies found that LTCI beneficiaries were satisfied with the pilot program.^40,42,43^ LTCI was associated with improvements of the caregivers’ (mainly family members) awareness rate of stress injury and pneumonia prevention as well as satisfaction.^41^Although the beneficiaries’ overall satisfaction was high, the satisfaction with long-term care activities that affect a certain degree of privacy (e.g., perineal cleaning, enema, catheterisation), clinical services and the professional skills of long-term care workers was relatively low.^42^

The questionnaire-based survey is the unanimous choice for studies reporting on satisfaction,^40–44^ and multiple surveys found that the influencing factors for satisfaction varied. A national-level survey conducted by Zhang et al. (2019),^40^ and another two district-level surveys conducted by Dai et al. (2019)^42^ and Zhang (2019)^43^ in Shanghai all found that living location was the most influential factor in satisfaction. The former study found that respondents living in Western cities reported higher levels of satisfaction than those living on the East coast, and the latter two found that the satisfaction level ranked in descending order from the urban areas, suburban areas to rural areas. This might be due to the imbalance in resources including facilities and trained professionals in each region. In addition to the living location, Zhang et al. (2019)^40^ found that family size also significantly affected the satisfaction level, where the family size is ranked in descending order from a family without children, family with one child and family with two or more children. Dai et al. (2019)^42^ found that education level, monthly pension level, marital status, and type of facility (i.e., home and community-based services or institutional long-term care services) had a significant influence on satisfaction.

Both Zhang et al. (2019)^40^ and Dai et al. (2019)^42^ found that gender, age, and degree of disability showed no significant association with satisfaction level. Additionally, Zhang et al. (2019)^40^ found that choices of care and monthly income did not correlate significantly with satisfaction, while Dai et al. (2019)^42^ found that occupation did not correlate significantly with satisfaction.

## DISCUSSION

Although almost all the studies found that LTCI in China was viewed positively, there remains limited evidence of the impact of LTCI in China, with only nine studies meeting the eligibility criteria to be included in this review. These studies found that 1) LTCI was associated with improvement of health conditions, including physical and mental conditions for the beneficiaries; 2) LTCI was correlated with a reduction in healthcare utilisation and expenditures; 3) LTCI was also correlated with a reduction in informal care by replacing it with formal care (e.g., decreasing caregivers’ financial burden as well as the caregiving time); 4) the beneficiaries and their family members were mainly satisfied with LTCI.

Many countries around the world, such as the Netherlands in the 1960s, the United States in the 1970s, Germany in 1995, Japan in 2000 and South Korea in 2008, introduced a public or private LTCI system.^45–49^ Studies in these countries found that LTCI had a major impact on the beneficiaries’ health conditions, medical expense and utilisation, caregivers’ informal care burden, and satisfaction.^45^

In South Korea, studies have found that LTCI had a positive effect on reducing the beneficiaries and caregivers’ physical and mental health problems. For instance, several studies found that LTCI could delay older adults’ cognitive impairments and disability progression as well as reduce mortality (with the reduction level varying by income).^50–52^

Another important question is whether LTCI reduces healthcare utilisation and medical expenditures. An insufficient supply of long-term care services might lead to allocative inefficiency (i.e., hospital bed shortages and increased medical expenditures).^53,54^ Most studies have confirmed that LTCI could reduce the financial burden on the beneficiaries’ families and government health expenditures, and promote the utilisation of medical resources.^50,52,55–57^ However, some studies reported that LTCI could reduce total healthcare expenditures, but increase outpatient care utilisations and pharmaceutical expenditures.^58^ In addition, some studies proposed introducing social capital and strengthening the effective combination of private and public LTCI in order to release the pressure on government expenditures as the population ages.^48,59–62^

The care and services for older adults usually includes support for basic activities of daily living (e.g., eating, dressing, and using the toilet) and instrumental activities of daily living (e.g., preparing meals, housekeeping, and managing medications),^30^ and these are commonly provided in the form of either formal care or informal care. The reduction of informal care replaced by formal care under the LTCI system could help solve this dilemma. Some studies have found that informal nursing had a considerably negative short-term impact on the mental health of female caregivers and that the implementation of LTCI alleviated the nursing burden of the beneficiaries’ family caregivers.^63–65^ Studies from the United States and Canada also indicated that LTCI could effectively reduce the informal caregivers’ responsibilities.^66,67^ The LTCI system serves as a cost-saving alternative for many informal caregivers who are in the workforce and thus face the dual task of providing care and working, and thus LTCI assists caregivers in reducing family-work conflict to maintain work-life balance.^68,69^ Substituting informal care for formal care is influenced by various factors, including government subsidies, economic income status, and older adults’ preference.^70–73^

The satisfaction level with LTCI has also been examined in other countries. Satisfaction with LTCI has varied with the majority of nursing staff satisfied while other studies on LTCI identified family caregivers as dissatisfied and concerned with the sustainability of the system.^65,74^

In addition to LTCI’s impact on the beneficiaries’ health conditions, medical expense and utilisation, caregivers’ informal care burden, and satisfaction, some research compared the health-related outcomes of home care with institutional care to find which was more effective, focused on the impact of the LTCI on utilisation of specific disease such as dementia, and impact of the LTCI on financial security assessment.^73,75,76^

While the studies mentioned above examined the impact of LTCI, they were conducted outside China. Our review is the first study to systematically review the impact of the LTCI pilots in 15 Chinese cities. However, there are several limitations of this review. One of the most important limitations is that most studies included originated from the same city—Shanghai. In addition, there is also a limitation with regard to the research design of some of the studies included in this review. Many of the studies did not utilise a rigorous sampling design and likely were underpowered to assess the impact of LTCI on the studied outcomes. Furthermore, few studies used experimental or quasi-experimental approaches (e.g. instrumental variable approach) to assess causal relationships. Finally, because the introduction of LTCI for China is a new phenomenon, all studies examined in this review only evaluated the short-term impacts of LTCI, typically encompassing a timeline of 1–2 years. The sustainability of the outcomes—for instance, reductions in health expenditures over a longer period of time requires ongoing evaluation.

Overall, there is a need for more rigorous research (e.g. studies following the CONSORT checklist or CHEERS Checklist) to evaluate the impact of LTCI on health-related outcomes in China in order to fill existing knowledge gaps and inform policy makers on the future nationwide rollout of LTCI.^77,78^

## CONCLUSION

We found that the existing evidence of the impact of LTCI in China is limited. However, the few studies that have been conducted found that LTCI was associated with an improvement in quality of life and physical pain and a reduction in healthcare utilisation and expenditures. LTCI was also associated with a reduction in the time that informal caregivers spent caring for the beneficiary. Satisfaction with the program was generally high. Given the small number of studies identified and their methodological weaknesses, further rigorous research on the impact of LTCI in China is needed to inform the future expansion of the program.

## Supplementary Material

Appendix

## Figures and Tables

**Figure 1.**
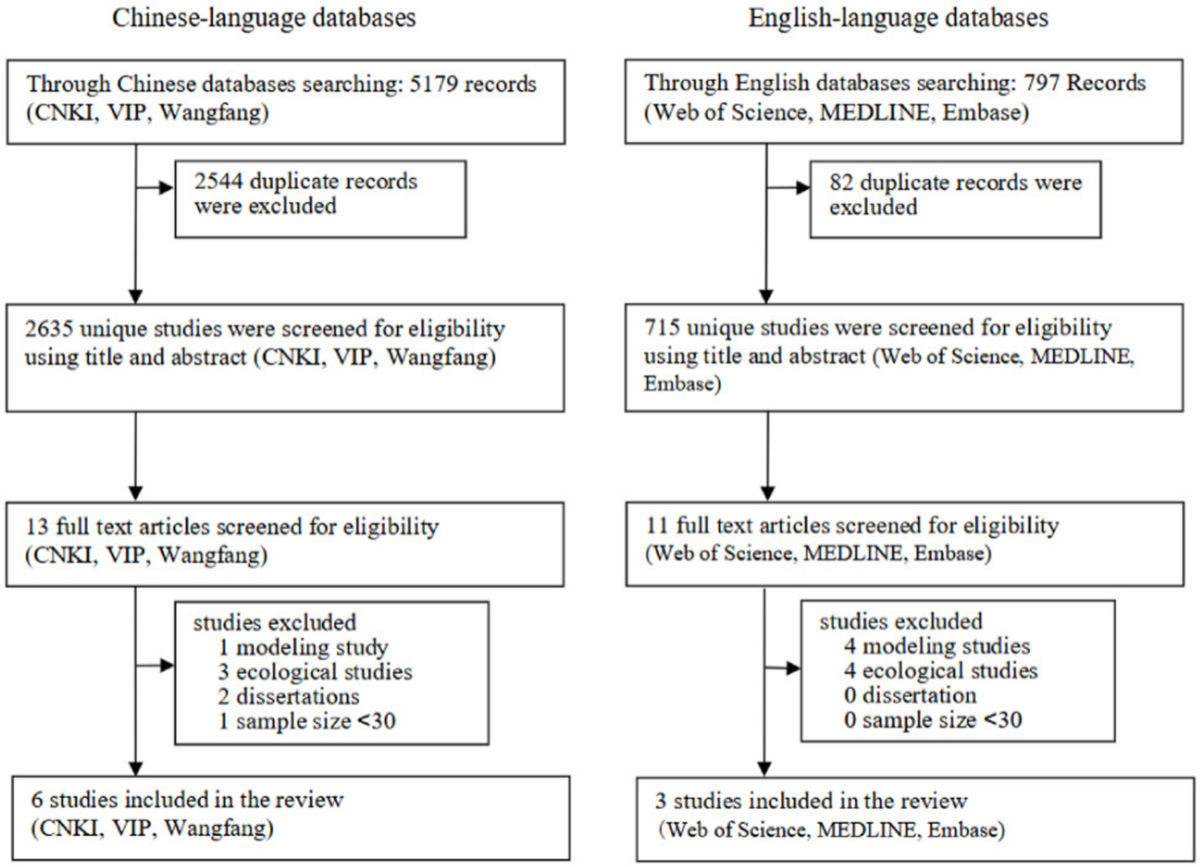
Systematic review study selection flow diagram CNKI=China National Knowledge Infrastructure

**Table 1.** Characteristics of included studies

		Study design (period)	Study setting	Study population	Sample size	Outcomes
Health-related outcomes	Yu et al., 2020 (C)^35^	Randomised controlled trial (2017–2018)	Shanghai city	Stroke patients in recovery	233 (intervention group: 112; control group: 111)	ADL—i.e., Barthel index
	Feng et al., 2020 (E)^36^	Difference in differences (2016–2017)	Shanghai city	Inpatients aged 60 and above enrolled in public health insurance (including UEBMI and URRBMI)	24,428 (15,986 in tertiary hospitals, 8442 in LTC facilities)	Hospital utilisation and expenditures
	Qi et al., 2019 (C)^37^	Cross-sectional study (2018)	Jinmen city, Hubei province	Disabled adults registered as permanent residents of Jinmen City	150	Disabled adults’ health expenditures
	Ma et al., 2019 (C)^38^	Difference in differences (2011, 2013, 2015)	Qingdao City, Shandong Province	Middle-aged and older adult (≥45 years old) residents	49,249	Residents’ outpatient expenses; mental and physical health status
Health-related expenditures	Qi et al., 2019 (C)^37^	Cross-sectional study (2018)	Jinmen city, Hubei province	Disabled adults registered as permanent residents of Jinmen City	150	Disabled adults’ health expenditures
Informal care	Zhang et al., 2020 (E)^39^	Cross-sectional study (2019)	Shanghai city	Families including an older adult who had used formal care provided by LTCI for 1 to 3 months (from 15 May 2019 to 15 August 2019) and a child who is primarily responsible for older adults’ daily informal care.	407	Families’ informal care burden
Satisfaction	Zhang et al., 2019 (E)^40^*	Cross-sectional study (2017)	15 pilot cities	Older adults aged 60 and above	1,500 (100 surveys for each city)	The willingness of Chinese citizens to formally expand the implementation of LTCI policy in China
	Chen et al., 2020 (C)^41$^	Cross-sectional study (Jan 2019-Dec 2019)	Jiuting town, Shanghai city	Older adults with an average age of 56.47 (±7.89) with a long-term care disability rating of level 5 or above	30	Family members’ knowledge and satisfaction
	Dai et al., 2019 (C)^42^*	Cross-sectional study (2018)	Xuhui, Putuo and Jinshan districts, Shanghai city	Older adults aged 60 and above receiving home and community-based services (HCBS) and institutional long-term care services	93	Older adults’ satisfaction
	Zhang, 2019 (C)^43^*	Cross-sectional study (2019)	Jingan, Changning and Qingpu districts, Shanghai city	Older adults in institutional long-term care facilities	243	Older adults’ satisfaction

* These studies only included people who have LTCI.

$ Only included people who have LTCI and their family members.

ADL=activities of daily living, UEBMI=Urban Employees Basic Medical Insurance, URRBMI=Urban and Rural Residents Basic Medical Insurance, LTC=long-term care, LTCI=long-term care insurance

**Table 2.** Summary of findings

		Findings	Evidence quality
Health-related outcomes	Yu et al., 2020 (C)^35^	LTCI had positive effects (e.g., decreasing caregivers’ burden, improving older stroke patients’ survival quality).	High
	Feng et al., 2020 (E)^36^	LTCI significantly reduced the length of stay, inpatient expenditures, and health insurance expenditures in tertiary hospitals by 41.0%, 17.7%, and 11.4%, respectively. A 1 yuan increase in LTCI expenditure will generate a 8.6 yuan decline in health insurance expenditures and 8.1% monthly decrease in outpatient visits in tertiary hospitals among people aged 80 years and above.	Medium
	Qi et al., 2019 (C)^37^	Out-of-pocket medical expenses per year were reduced after older adults were enrolled in LTCI.	Low
	Ma et al., 2019 (C)^38^	LTCI improved the beneficiaries’ mental health status and relieved their physical pain without hazard consequences; it also reduced the average outpatient expenses of the middle-aged and older residents in the pilot city by 210.51 yuan per month, and the average hospitalisation expenses were reduced by 1901.69 yuan per year.	Medium
Health-related expenditures	Qi et al., 2019 (C)^37^	Out-of-pocket medical expenses per year were reduced after older adults were enrolled in LTCI.	Low
Informal care	Zhang et al., 2020 (E)^39^	With LTCI there was an average of 12.36h less of informal care performed weekly, including household activities of daily living (HDL) tasks, activities of daily living (ADL) tasks, instrumental activities of daily living (IADL) tasks and supervision tasks. Although the reduction of total informal care time varied according to care recipients’ gender and health status, each additional hour of formal care generally reduced 0.473h of informal care.	Low
Satisfaction	Zhang et al, 2019 (E)^40^	The satisfaction rate towards LTCI was 72.24%, with 20% being dissatisfied with the policy and 8% neutral. Living location and family size significantly influenced the level of satisfaction, while other factors showed no significance, including gender, age, degree of disability, choices of care, and monthly income.	Low
	Chen et al., 2020 (C)^41^	With LTCI, the caregivers’ awareness of stress injury and pneumonia prevention, as well as the degree of satisfaction, were all significantly improved.	Low
	Dai et al., 2019 (C)^42^	The beneficiaries’ overall satisfaction was high; however, the satisfaction towards long-term care activities that affect a certain degree of privacy (e.g., perineal cleaning, enema, catheterisation), clinical servicess, and long-term care workers’ professional skills was relatively low. Living location was the most influential factor towards satisfaction; education level, monthly pension level, marital status, and types of facilities (i.e., home and community-based services or institutional long-term care services) had a significant influence on the satisfaction, while gender, age, occupation, and degree of disability showed no significant association with satisfaction level.	Low
	Zhang, 2019 (C)^43^	Living location was the most influential factor in satisfaction, ranking in descending order from urban areas and suburban areas to rural areas. This might be due to the imbalance of resource allocation, including facilities and trained professionals in each region.	Low
